# An Effect of Chronic Stress on Prospective Memory via Alteration of Resting-State Hippocampal Subregion Functional Connectivity

**DOI:** 10.1038/s41598-019-56111-9

**Published:** 2019-12-23

**Authors:** Jierong Chen, Zhen Wei, Hongying Han, Lili Jin, Chuanyong Xu, Dan Dong, Jianping Lu, Guobin Wan, Ziwen Peng

**Affiliations:** 1 Department of Child Psychiatry and Rehabilitation, Affiliated Shenzhen Maternity & Child Healthcare Hospital, Southern Medical University, Shenzhen, China; 2 Center for Studies of Psychological Application, School of Psychology, South China Normal University, Guangzhou, China; 3 Guangdong Key Laboratory of Mental Health and Cognitive Science, South China Normal University, Guangzhou, China; 4 Department of Psychiatry, the Third Affiliated Hospital, Sun Yat-Sen University, Guangzhou, China; 5 Department of Child Psychiatry, Shenzhen Kangning Hospital, Shenzhen University School of Medicine, Shenzhen, China

**Keywords:** Learning and memory, Learning and memory, Psychology, Psychology

## Abstract

The alteration of hippocampal function by chronic stress impairs higher order cognitive functions such as prospective memory (PM). However, how chronic stress affects hippocampal subregions related to PM remains largely unknown. In this study, the altered functional network of hippocampal subregions related to PM in chronic stress was explored. College students (*N* = 21) completed PM tasks and resting-state functional magnetic resonance imaging scans one month prior to (baseline) and during the final examination week (chronic stress). Hippocampal subregions’ seed-based functional connectivity (FC) and PM were compared between baseline and chronic stress. PM performance declined in chronic stress. The FC of the cornu ammonis 2, 3 and dentate gyrus (CA23DG) with the bilateral caudate and precuneus was increased in chronic stress, while the FC of the subicular complex (SUBC) with the left middle frontal gyrus, the left inferior parietal gyrus and the right supramarginal gyrus was decreased. There was a negative correlation between PM performance and the FC of hippocampal subregions. We found chronic stress impairs PM by decreasing the FC of SUBC and increasing the FC of CA23DG. These findings suggest functional changes in hippocampal subregion networks as a mechanism underlying the impairment of PM in chronic stress.

## Introduction

Chronic stress can be defined as long-term exposure to repeated experiences perceived to be stressful^1^. Coincident with economic development, chronic stress has increased in modern society, and it has a long-term impact on mental health^2^. Numerous studies have indicated that chronic stress impairs high-order cognitive function as exemplified by impairment of prospective memory (PM)^3–5^. Previous studies found that PM is sensitive to stress^6–8^. Exposure to chronic stress leads to activation of hypothalamic-pituitary-adrenocortical (HPA) axis and the release of glucocorticoids^9^. These bind to receptors in specific areas of the brain, such as the hippocampus, a region critical for PM^10^. A set of recent neuroimaging studies found that acute stress altered cognition-related activity in specific brain regions^11,12^. However, the effect of chronic stress on PM related to hippocampual function in humans remains unclear.

PM is a form of memory that involves remembering to perform a planned action or recall a planned intention that is supported by the frontoparietal network^13,14^. There are two kinds of prospective memory: time- and event-based PM (TBPM and EBPM, respectively)^15,16^. Previous study found the commonalities and differences in the neural substrates of EBPM and TBPM^17^. Planned information is stored in the hippocampus, as a buffer of memory information^18^. Previous studies have indicated that the hippocampus is one of the crucial neural substrates of PM^10^. Exposure to chronic stress leads to reversible impairment of hippocampus morphology, accompanied by dysfunction of PM^19,20^. Empirical evidence from multiple pharmacological studies in animals has shown that chronic stress has different effects on different hippocampal subregions, such as neuronal atrophy in CA3 and change of GABAergic inhibition in CA1^21,22^. In sum, chronic stress appears to impair the function and/or morphology of the hippocampus related to PM. However, the potential relationship between hippocampal subregions and different kinds of prospective memory in chronic stress remains unclear.

The hippocampus is a crucial subcortical structure for rapid acquisition and persistence of memories^10^. Chronic stress activates the HPA axis, releasing corticosteroids, for which the hippocampus contains the highest density of receptors^23^. Besides, chronic stress alters structure and/or neurotransmitter metabolism in hippocampus related to changes of its neurocircuitry, with implications for its stress-associated dysfunctions^24,25^. Hence, the hippocampus is sensitive to stress through the effect of corticosteroids, which have a significant impact on its functions, such as PM^26,27^. Importantly, the hippocampus is a heterogeneous brain region that can be divided into three subregions: the cornu ammonis (CA1, CA2, and CA3), the dentate gyrus (DG), and the subicular complex (SUBC)^28^. On one hand, according to recent research, hippocampal subregions may be involved in different cognitive processes within the memory system^29–32^. For instance, Suthana found that the CA2, CA3, and dentate gyrus (CA23DG) function to code new associations with novel information, while the SUBC functions to retrieve learned associations^33^. Chronic stressors also elicit subregion specific responses^34^. Previous studies found that chronic stress causes atrophy of CA3 pyramidal neurons but not in the DG^35^. According to the above findings, chronic stress may cause specific effect on different hippocampal subregion function and/or structure which are involved in the processing of PM.

In the present study, we chose final examinations as a chronic stressor for academic performance from a few days of written exams is the primary factor determining success for freshman in Chinese college^36^. Performance on final examinations affects scholarship applications and postgraduate recommendations. Hence, college students spend a lot of time preparing for examinations and are inevitably in a state of stress during this period^37^. In general, there are seven or eight written examination in one week at the end of the term.

We used resting-state functional magnetic resonance imaging (fMRI) to investigate how chronic stress alters the hippocampal subregion network related to PM. We hypothesized that (1) participants would score lower on PM tasks in chronic stress, (2) there would be significant changes in the functional connectivity (FC) of hippocampal subregions under chronic stress compare to baseline, and (3) PM performance would be selectively associated with changes in the FC of different hippocampal subregions in chronic stress.

## Results

### Sample description

The demographic characteristics are shown in Table 1. Twenty-one college students were recruited in our study (11 male, 10 female; age = 19.89 ± 0.83 years). They came from different majors, including Psychology, Fine Art, Politics and Music. The intervals between the two time points was 31.25 ± 2.67 days (Table 1).

**Table 1:** Demographic and neuropsychological data in baseline and chronic stress.

	Baseline	Chronic stress	t	*p*
Number	21			
Gender(male:female)	11:10			
Age	19.89 ± 0.83			
Time interval	31.25 ± 2.67			
IQ	108.67 ± 10.63			
Frustration-SLSI	15.60 ± 3.44	16.85 ± 3.12	1.518	0.146
Conflict-SLSI	7.10 ± 2.42	8.40 ± 2.06	2.762	0.012
Stress-SLSI	11.50 ± 2.44	13.00 ± 2.08	1.770	0.093
Change-SLSI	7.65 ± 1.35	9.10 ± 1.62	3.454	0.003
Self-reinforcing-SLSI	16.45 ± 4.15	17.10 ± 4.13	0.555	0.585
Physiological reaction-SLSI	20.15 ± 4.08	23.65 ± 5.92	2.056	0.054
Emotional reaction-SLSI	8.30 ± 2.30	10.35 ± 3.38	2.053	0.054
Behavior reaction-SLSI	11.15 ± 2.58	12.35 ± 3.44	1.156	0.262
Cognitive reaction-SLSI	6.10 ± 1.41	6.40 ± 1.35	0.688	0.500
Total scores-SLSI	105.25 ± 14.69	116.50 ± 17.52	2.864	0.010
EBPM	0.93 ± 0.12	0.76 ± 0.26	−2.719	0.013
TBPM	1.00 ± 0.00	0.62 ± 0.27	−6.921	0.000

Notes: Data are presented as mean ± SD. Abbreviations: SD, standard deviation; SLSI, Student-Life Stress Inventory; EBPM, event-based prospective memory; TBPM, time-based prospective memory.

Significantly different from baseline (p < 0.05, two-tailed test).

### Neuropsychological scores

The average SLSI score was significantly increased in chronic stress compared to baseline (Conflict-SLSI: *t* = 2.762, *p* = 0.012; Change-SLSI: *t* = 3.454, *p* = 0.003; Total scores-SLSI: *t* = 2.864, *p* = 0.010) (Table 1). The IQ (intelligence quotient) of participants was 123.67 ± 10.63 (Table 1).

### Performance of PM

Compared to baseline, prospective memory task scores significantly decreased in chronic stress (TBPM: *t* = 0.00, *p* = −6.06, EBPM: *t* = 0.04, *p* = −2.15) (Table 1). As shown in Table 2, these was significant difference in difference of EBPM and TBPM between baseline and chronic stress (*t* = 3.342, *p* = 0.002).

**Table 2:** Performance and difference of PM in baseline and chronic stress.

	EBPM	TBPM	*t*	*p*
Baseline	0.93 ± 0.12	1.00 ± 0.00		
Chronic stress	0.76 ± 0.26	0.62 ± 0.27		
Difference	0.11 ± 0.22	0.38 ± 0.26	3.342	0.002

Notes: Data are presented as mean ± SD. Abbreviations: SD, standard deviation; EBPM, event-based prospective memory; TBPM, time-based prospective memory.

Significantly different from baseline (p < 0.05, two-tailed test).

### Hippocampal subregion network within-group analysis

**Figure 1:**
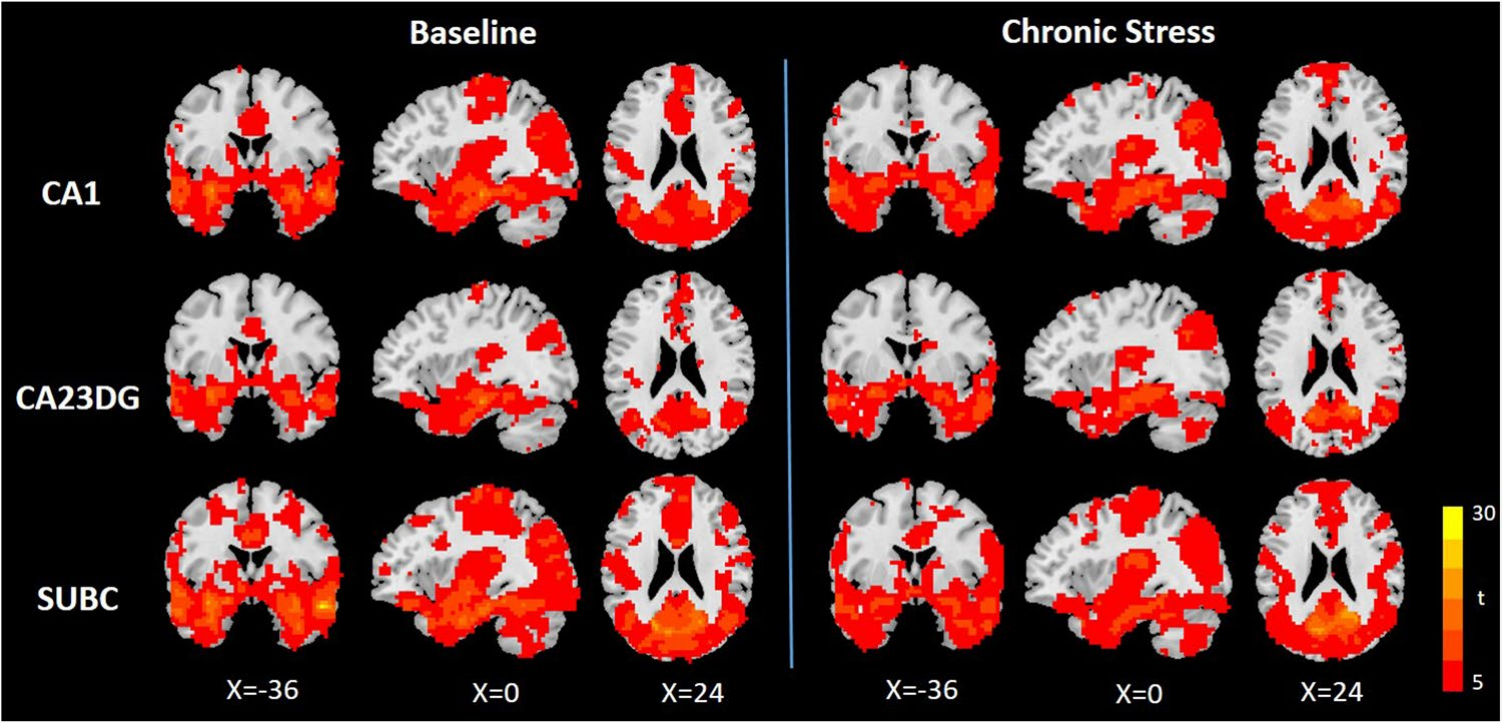
Functional connectivity pattern of hippocampal subregion networks in baseline and chronic stress. Statistical significance was set at p < 0.0001, corrected by FDR. Abbreviations: CA1, Cornu ammonis1; CA23DG, Cornu ammonis 2, 3 and dentate gyrus; SUBC, subicular complex.

The patterns of each hippocampal subregion FC network at baseline and under chronic stress are illustrated in Fig. 1. In both conditions, each functional network of hippocampal subregions involved diffuse subcortical, medial frontal, temporal cortical, parietal and cerebellar sites (*p* < 0.0001, corrected by FDR). The patterns of FC were similar to the network connections observed in previous studies on the whole and bilateral hippocampus^38,39^. However, in both chronic stress and baseline conditions, the stronger FC of hippocampal subregional seeds was located in the temporal lobe. In addition, regions that were close to the seed region showed stronger connectivity than other regions in each of the three hippocampal subregions.

### Longitudinal changes in hippocampal subregion networks

**Figure 2:**
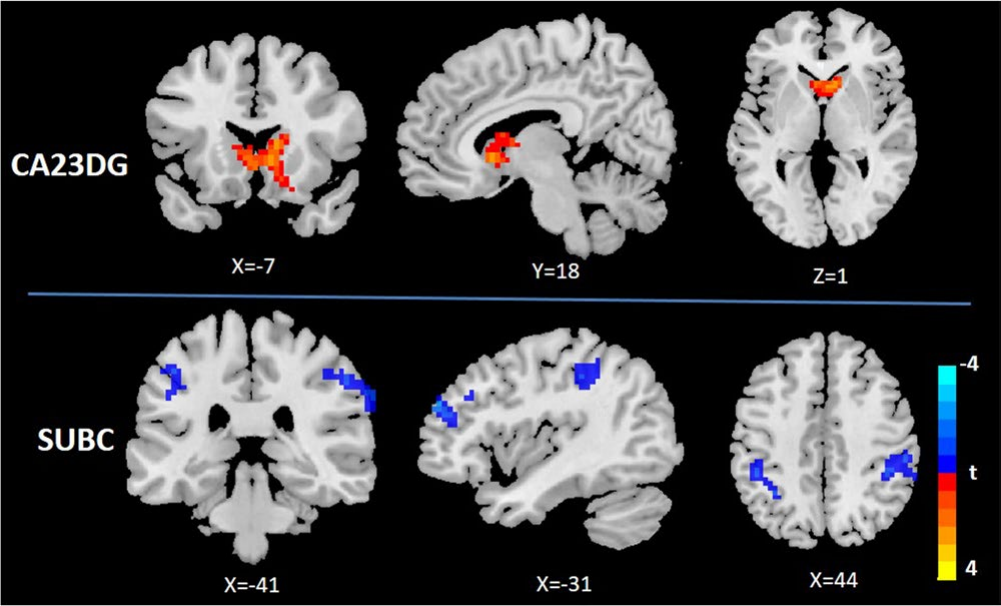
Longitudinal changes in CA23DG and SUBC hippocampal subregional networks. Compared to baseline, (1) the CA23DG subregion showed increased FC with bilateral caudate and precuneus during chronic stress, (2) the SUBC subregion showed decreased FC with the left middle frontal gyrus, left inferior parietal gyrus and right supramarginal gyrus during chronic stress. Significance threshold was set at p < 0.05, AlphaSim corrected. Abbreviations: CA23DG, Cornu ammonis 2, 3 and dentate gyrus; SUBC, subicular complex.

As shown in Fig. 2, the CA23DG showed greater FC with the bilateral caudate and precuneus in chronic stress compared to baseline. In addition, the SUBC showed decreased FC with the left middle frontal gyrus, left inferior parietal gyrus, and right supramarginal gyrus in chronic stress compared to baseline (Fig. 2). There was no significant change in the CA1 network.

### Correlations among hippocampal subregional networks, SLSI scores and PM performance in chronic stress

**Figure 3:**
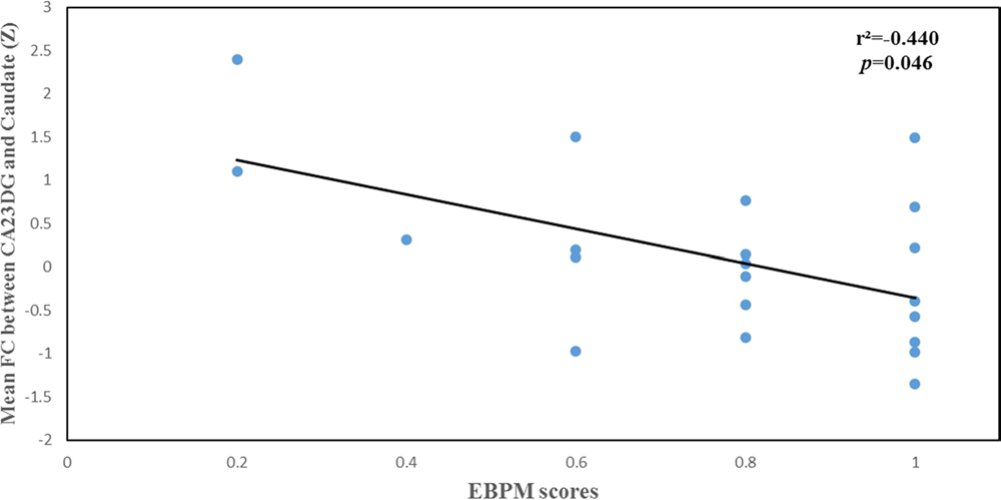
The association between mean functional connectivity of CA23DG-Caudate and performance of event-based prospective memory in chronic stress. Abbreviations: CA23DG: Cornu ammonis 2, 3 and dentate gyrus.

**Figure 4:**
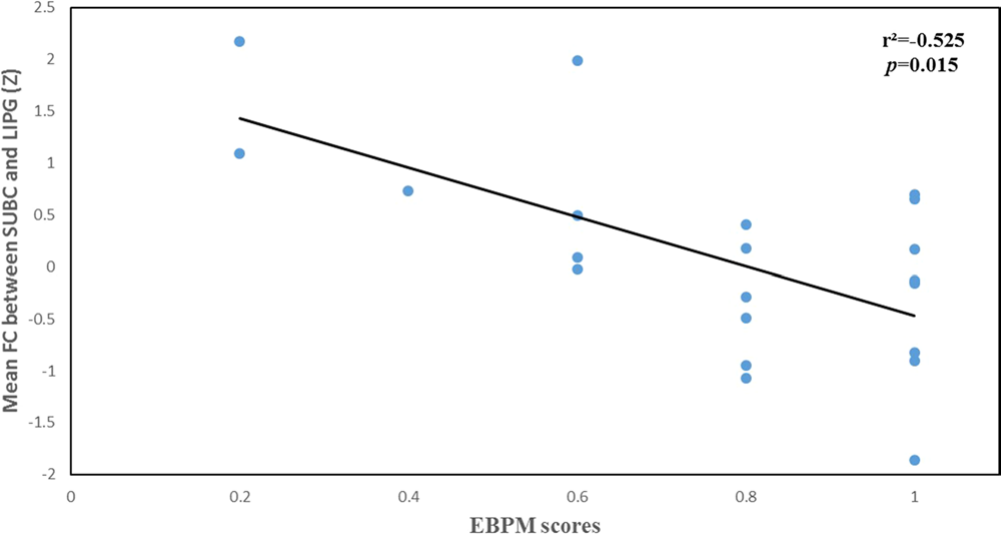
The association between mean functional connectivity of SUBC-LIPG and performance of event-based prospective memory in chronic stress. Abbreviations: SUB, subicular complex; LIPG, left inferior parietal gyrus.

**Figure 5:**
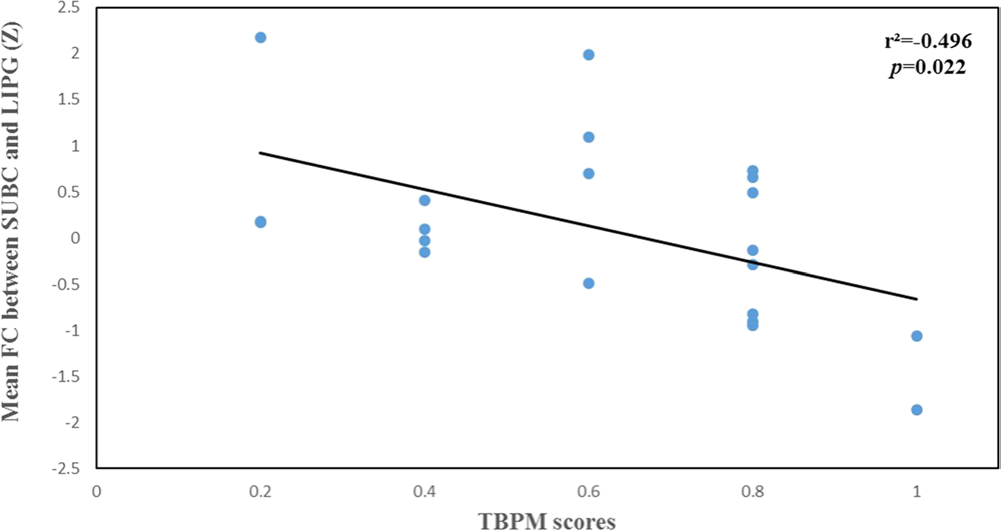
The association between mean functional connectivity of SUBC–LIPG and performance of time-based prospective memory in chronic stress. Abbreviations: SUB, subicular complex; LIPG, left inferior parietal gyrus.

As shown in Table 3, the FC between the bilateral caudate and the CA23DG was positively correlated with SLSI scores (Conflict-SLSI: *r* = 0.616, *p* = 0.011; Stress-SLSI: *r* = 0.582, *p* = 0.018; Change-SLSI: *r* = 0.543, *p* = 0.030; Emotional reaction-SLSI: *r* = 0.571, *p* = 0.021; Total scores- SLSI: *r* = 0.667, *p* = 0.005) (Table 3). The FC between the left inferior parietal gyrus and the SUBC was negatively correlated with the SLSI scores (Physiological reaction: *r* = −0.487, *p* = 0.047) (Table 3). A significant negative correlation was also found between the FC of hippocampal subregional networks and PM performance in chronic stress. Particularly, the FC between the bilateral caudate and the CA23DG was negatively correlated with EBPM performance (*r* = −0.440, *p* = 0.046; Fig. 3). The FC between the left inferior parietal gyrus and the SUBC was negatively correlated with EBPM performance (*r* = −0.525, *p* = 0.015; Fig. 4). In addition, the FC of the SUBC with the left inferior parietal gyrus was negatively correlated with TBPM performance (r = −0.496, *p* = 0.022; Fig. 5). There was no significant result with regard to the moderating effect.

**Table 3:** The correlation between SLSI and FC of hippocampal subregions in chronic stress.

	CA23DG-Caudate		SUBC-LIPG	
	r	*p*	r	*p*
Frustration-SLSI	0.390	0.135	−0.252	0.328
Conflict-SLSI	0.616	0.011	−0.028	0.914
Stress-SLSI	0.582	0.018	0.147	0.573
Change-SLSI	0.543	0.030	−0.036	0.890
Self-reinforcing-SLSI	0.394	0.131	−0.407	0.105
Physiological reaction-SLSI	0.341	0.196	−0.487	0.047
Emotional reaction-SLSI	0.571	0.021	−0.241	0.350
Behavior reaction-SLSI	0.511	0.043	−0.232	0.371
Cognitive reaction-SLSI	0.336	0.203	−0.012	0.963
Total scores-SLSI	0.667	0.005	−0.388	0.124

Notes: Data are presented as mean ± SD. Abbreviations: SD, standard deviation; SLSI, Student-Life Stress Inventory; FC, functional connectivity; CA23DG, Cornu ammonis 2, 3 and dentate gyrus.

Significant result are bolded at P < 0.05.

## Discussion

In this study, we aimed to investigate chronic-stress-induced modulations in the activity of PM-related hippocampal subregions. The results confirmed our hypothesis regarding the altered FC of hippocampal subregion networks in chronic stress. Our results showed that (a) participants scored lower on the PM task in chronic stress compared to baseline; (b) the CA23DG FC network showed significantly increased FC with the bilateral caudate and precuneus, while the SUBC network showed significantly decreased FC with the left middle frontal gyrus, left inferior parietal gyrus and right supramarginal gyrus in chronic stress; and (c) the altered FC of hippocampal subregions was significantly associated with PM performance in chronic stress. These results may provide insight into the underlying neural mechanism of PM impairment related to hippocampal subregions in chronic stress.

During examination week, participants showed higher stress levels compared to baseline. This finding is consistent with previous studies, which showed that academic examinations are a key factor stress-causing for Chinese students^36,37^. In addition, we found a correlation between SLSI scores and the FC of hippocampal subregions. This indicated that the altered FC of hippocampal subregions may be related to increased stress levels. Consist with previous study that chronic stress causes hippocampal morphological changes and impairs hippocampal function^35^. In the present study, participants had lower scores on TBPM and EBPM during chronic stress compared to baseline. A previous study indicated that chronic stress affects cognitive function including PM^4^. Interestingly, although both EBPM and TBPM were affected by stress in our study, TBPM was affected to a greater extent compared to EBPM. These finding suggest that TBPM is more sensitive to chronic stress than EBPM. This is in agreement with other studies that have found TBPM to be more affected by emotional factors than EBPM^8^. Moreover, our results support Ellis and Ashbrook’s (1988) model suggesting that emotional factors adjust the allocation of cognitive resources, which are more necessary in TBPM tasks than EBPM tasks^15,40,41^.

We found an alteration of the intrinsic FC network of CA3DG and SUBC, accompanied by impairment of PM, in chronic stress. Previous studies have indicated that stress activates the HPA, releasing stress-related hormones that bind to receptors in hippocampus, which affects memory-related function^42^. Our study found opposite FC activation patterns in different hippocampal subregions, with activated FC of the CA23DG network and deactivated FC of the SUBC network. Numerous animal studies have shown that stress has distinct impacts on different hippocampal subregions which may involve in different cognitive processes^35,43^. Moreover, previous studies reported functional dissociation in hippocampal subregions^33^. This study concurred with those findings and extended the knowledge by demonstrating chronic stress modulates the FC of hippocampal subregions.

In the current study, the FC of the CA23DG with the caudate was negatively correlated with EBPM scores. Previous studies have shown that TBPM and EBPM performance depends on shared and distinct cognitive abilities that rely on specific brain regions^17^. The caudate nucleus, which provide an early analysis of the affective properties of the stimuli in a PM task, is activated by aversive stimuli^44^. Performance on EBPM tasks is largely dependent on the cognitive functions of working memory and executive function, which the caudate nucleus is critically involved in^45–49^. Therefore, these findings further suggest that altered cooperation of the CA23DG subregion with the caudate nucleus may be a key factor in EBPM under chronic stress.

In chronic stress, the FC of the SUBC with the left inferior parietal gyrus was negatively correlated with both EBPM and TBPM scores. This result suggests that the deactication of the FC of the SUBC and the inferior parietal gyrus under chronic stress related to PM impairment. PM success is linked to the anterior prefrontal cortex, parietal lobe, and hippocampus regions^50,51^. Previous studies have confirmed that the parietal cortex is a shared substrate of EBPM and TBPM that maintains future intention^17^. Other studies found that the parietal lobe plays an important role in attention and memory retrieval, which, in turn, play an important role in PM^52,53^. Furthermore, deactivation of inferior parietal gyrus induced by medicine was found to increase failures of PM^54^. To summarize, the altered FC of the SUBC and left inferior parietal gyrus in our study may reflect the underlying neurological mechanism whereby chronic stress impairs PM.

This study has two limitations: First, our study had a relatively small sample size. Further studies with larger sample sizes with a more diverse age range are needed. Second, we did not include stress-sensitive psychophysiological measurements, such as cortisol and heart rate. In future studies, the stress level of participants will be measured in different ways, including psychophysiological assessment and self-assessment questionnaire.

To our knowledge, this is the first study to explore the chronic stress-induced specific activation pattern of hippocampal subregions related to impairment of PM. The results suggest that the activation of the FC of SUBC and CA23DG under chronic stress related to impairment of EBPM, while the deactivation of the FC of SUBC is related to both EBPM and TBPM. These findings suggest functional changes in hippocampal subregion networks as a mechanism underlying the impairment of PM in chronic stress.

## Methods

### Participants

One hundred and five freshmen were recruited from South China Normal University. All participants were right-handed and were selected according to the following criteria: (1) age between 19 and 21 years old, (2) moderate stress as indicated by a the Student-Life Stress Inventory (SLSI) score of >79 and <153, (3) more than 12 years of education, (3) no history of neurological, or psychiatric disorder or head injury, and (4) no history of alcohol/drug dependence. Finally, 25 students (13 male, 12 female) with moderate stress scores were selected as participants. Data were collected at two time points from the participants in May, 2016 (one month prior to examine week; baseline) and June, 2016 (examine week; chronic stress). Informed written consent was obtained from all subjects who participated in the current study. The study was approved by the Research Ethics Board of the South China Normal University. The experiments were performed in accordance with the approved guidelines.

### Neuropsychological assessment

The SLSI is a self-administered 51-item Likert-type questionnaire that requires participants to rate their life stress from 1 to 5 (1 = never, 2 = seldom, 3 = occasionally, 4 = often, and 5 = most of the time). The SLSI consists of two parts: stressors and reactions to stressors. The stressors measurement consists of five sections frustrations, conflicts, pressures, changes, and self-imposed stressors while reactions to stressors includes physiological, emotional, behavioral, and cognitive sections. A greater level of stress is indicated by higher scores on stressors and reaction to stress. The SLSI has been shown to have satisfactory reliability and high internal consistency (Cronbach’s α = 0.92)^55^.

Estimated IQ was assessed with the Wechsler Adult Intelligence Scale-Revised (WAIS-R), and subjects with a total score of less than 80 were excluded^56^. WAIS-R was performed by experienced clinical psychologists.

### Prospective memory assessment

PM performance was measured through a classical dual-task paradigm^15^ including an ongoing task and a prospective memory task. Ongoing tasks are same in EBPM and TBPM, but the PM task is different. The PM task was divided into two categories: a time-based prospective memory task (TBPM task) and an event-based prospective memory task (EBPM task).

#### Ongoing-task

For the ongoing task, four Chinese words were displayed in the center of a screen (e.g., 安居乐业). The subject was asked to judge whether the four words formed an idiom. Subjects were instructed to press the “F” key if the four words did not form an idiom (e.g., 直接选举) and the “J” key if they did (e.g., 大材小用).

#### PM task

In the EBPM task, subjects were instructed to press the space bar if they detected the name of an animal (target event) within the four words (e.g. 如鱼得水). There were five target words (e.g., 虎,马,鼠,鱼,鸡), which appeared at approximately one minute intervals. Non-animal words, such as colors and numbers were used as interference. The participants received one point for each correct response to a target event (total of five target events). Therefore, scores ranged from 0 to 5.

In the TBPM task, a standard clock was placed in the lower left corner of the screen. Subjects were instructed to monitor time throughout the trial and to press the spacebar after one minute. The task lasted for approximately 5 minutes. The participants received one point for each correct response after a full minute. Again, scores ranged from 0–5.

### fMRI data acquisition

fMRI data were collected by a clinically approved Siemens Magnetom Avanto 3.0 T (Simens Medical Solutions, Erlangen, Germany) using the Siemens 12-channel receive-only head coil. The imaging sessions included structural T1, a resting condition FC, and two task-related functional acquisitions. Sessions were conducted on the same day and the Siemens Auto Align scout protocol was used to minimize variations in head positioning. For structural analysis, a T1 high-resolution anatomical sequence, 3D MPRAGE (magnetization prepared rapid gradient echo) was performed with the following scan parameters: repetition time (TR) = 2.4 s, echo time (TE) = 3.62 ms, 160 sagittal slices with no gap, field-of-view (FoV) = 234 mm, flip angle (FA) = 8°, in-plane resolution = 1.2 × 1.2 mm² and slice thickness = 1.2 mm. During resting-condition fMRI acquisition, a gradient T2 weighted echo-planar imaging (EPIs) protocol was used and participants were instructed to keep their eyes closed and to think about nothing in particular. The imaging parameters were: 100 volumes, TR = 3 s, TE = 50 ms, FA = 90°, in-plane resolution = 3.4 × 3.4 mm², 30 interleaved slices, slice thickness = 5 mm, imaging matrix of 64 × 64 and FoV = 220 mm. fMRI was acquired using: TR = 2 s, TE = ms, FA = 90°, in-plane resolution and slice thickness = 3.3 mm, 38 ascending interleaved axial slices with no gap and FoV = 212 mm. The functional paradigm protocol was previously described and the paradigm was presented using the fully integrated fMRI system IFIS-SA^57^.

### Image pre-processing

Prior to data processing and analysis, all images were visually inspected to confirm the absence of head motion and brain lesions. Pre-processing was conducted with Statistical Parametric Mapping (SPM12, http://www.fil.ion.ucl.ac.uk/spm), Data Processing Assistant for Resting-Condition fMRI (DPARSF 4.3 Advanced Edition), Resting-Condition fMRI Data Analysis Toolkit (REST 1.8, http://www.restfmri.net) and MATLAB 8.30. The first 10 volumes of the scanning session were discarded to achieve signal stabilization and allow participants to adjust to the scanner noise. Scans with head motion of more than 3 mm maximum displacement in x, y, or z direction or 3° of any angular motion were excluded from analysis. The remaining images were spatially normalized to the standard MMI (Montreal Neurological Institute) echo-planar imaging template, resampled to 3 × 3 × 3 mm³ cubic voxels, and smoothed with a Gaussian kernel 6 × 6 × 6 mm³. To further reduce the effects of confounding factors, the white matter (WM) signal, the cerebrospinal fluid (CSF) signal and six motion parameters were regressed out from the data. Finally, the images were temporally band-pass filtered (0.01–0.08 Hz) and liner trends were removed.

Data from four subjects with poor quality images and head movement more than 3 mm or 3 degree in any direction were excluded from analysis. The final analysis included data from 21 participants.

### FC analysis

**Figure 6:**
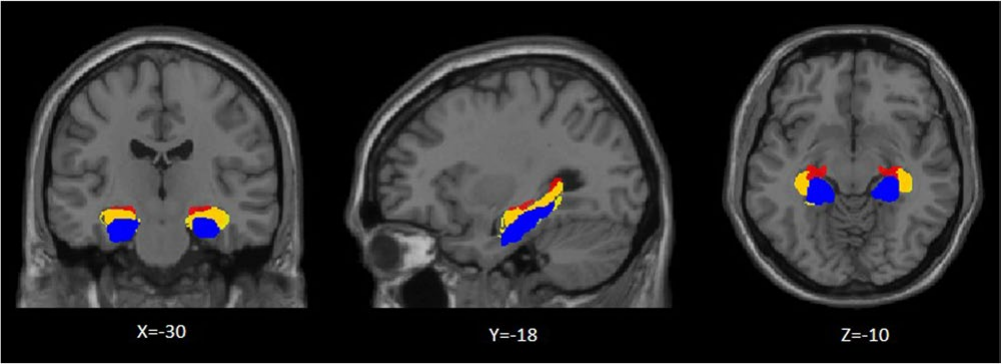
Location of hippocampal subregions. Yellow: CA1 (Cornu ammonis1); Red: CA23DG (Cornu ammonis 2, 3 and dentate gyrus); Blue: SUBC (subicular complex).

The Anatomy toolbox in SPM12 (http://www.fz-juelich.de/inm/inm-1/DE/Forschung/docs/SPMAnatomyToolbox/SPMAnatomyToolbox_node.html) was used to identify the three hippocampal subregions (CA1, CA23DG, SUBC) (Fig. 6). Regions of interest were defined according to previous studies^58,59^. For each subject, a voxel-wise-based FC was computed separately for each hippocampal subregion using the Data Processing Assistant for Resting-State fMRI (dparsf, http://www.rest.restfmri.net). Cross-correlation values between each subregion and the rest of the brain were then calculated. A Fisher transformation was applied to improve the normality of the correlation coefficient^60^.

### Statistical analysis

A within group analysis was performed to explore differences in the spatial maps of FC in baseline and chronic stress using one-sample t-tests. Data were corrected for false discovery rate (FDR), and the statistical threshold was set at *p* < 0.0001. To avoid potential interpretational confounds of negative connectivity caused by correction for the global signal, only positive functional connectivity was examined^61^.

A between-group analysis was performed to detect changes in PM (TBPM and EBPM) and FC in different hippocampal subregions between chronic stress and baseline (CA1, CA23DG, and SUBC subregion network analysis were performed separately) using paired *t* tests. Statistical significance was set at *p* < 0.05 using AlphaSim correction. For measuring extent which chronic stress impact, a between-group analysis was performed to compare the difference of TBPM and EBPM using two-sample t-test.

The relationships between FC of hippocampal subregions and SLSI and PM scores were explored. The mean FC strengths of the clusters showing significant between-group differences were extracted in chronic stress. Spearman’s correlation analysis was performed to investigate the relationship between PM and the FC of hippocampal subregions. A partial correlation analysis was performed to explore the relationship between the FC of hippocampal subregions and SLSI scores, controlling for covariates like gender.

We further explored whether the FC of hippocampal subregions moderates the relationship between the stress level and PM. The SPSS PROCESS macro program, designed by Hayes^62^, was used to measure the mediating or moderating effect. Within PROCESS, model 1 was selected and the confidence interval was set to 95%. In the moderation models, the SLSI scores were entered as the predictor (X), PM performance as the outcome (Y), and mean FC of hippocampal subregions as the moderator (M). All statistical tests were evaluated at the *p* < 0.05 significance level and constituted two-tailed tests.
